# Concurrent Validity and Reliability of Two Portable Powermeters (Power2Max vs. PowerTap) to Measure Different Types of Efforts in Cycling

**DOI:** 10.3390/s23187745

**Published:** 2023-09-07

**Authors:** Javier Iglesias-Pino, Alba Herrero-Molleda, Miguel Ángel Saavedra-García, Juan García-López

**Affiliations:** 1 Human Movement and Sports Performance Analysis (AMRED), Faculty of Physical Activity and Sports Sciences, Universidad de León, 24071 León, Spain; javi_ipin@hotmail.es (J.I.-P.); juan.garcia@unileon.es (J.G.-L.); 2 Grupo de Investigación en Ciencias del Deporte (INCIDE), Departamento de Educación Física y Deportiva, Universidade da Coruña, 15179 A Coruña, Spain; miguel.saavedra@udc.es

**Keywords:** road cycling, monitoring, power output, pedaling rate

## Abstract

The purpose was to assess the concurrent validity and reliability of two portable powermeters (PowerTap vs. Power2Max) in different types of cycling efforts. Ten cyclists performed two submaximal, one incremental maximal and two supramaximal sprint tests on an ergometer, while pedaling power and cadence were registered by both powermeters and a cadence sensor (GarminGSC10). During the submaximal and incremental maximal tests, significant correlations were found for power and cadence data (r = 0.992–0.997 and 0.996–0.998, respectively, *p* < 0.001), with a slight power underestimation by PowerTap (0.7–1.8%, *p* < 0.01) and a high reliability of both powermeters (*p* < 0.001) for measurement of power (ICC = 0.926 and 0.936, respectively) and cadence (ICC = 0.969 and 0.970, respectively). However, during the supramaximal sprint test, their agreement to measure power and cadence was weak (r = 0.850 and −0.253, *p* < 0.05) due to the low reliability of the cadence measurements (ICC between 0.496 and 0.736, and 0.574 and 0.664, respectively; *p* < 0.05) in contrast to the high reliability of the cadence sensor (ICC = 0.987–0.994). In conclusion, both powermeters are valid and reliable for measuring power and cadence during continuous cycling efforts (~100–450 W), but questionable during sprint efforts (>500 W), where they are affected by the gear ratio used (PowerTap) and by their low accuracy in cadence recording (PowerTap and Power2Max).

## 1. Introduction

Portable powermeters are devices designed to measure the power output (i.e., the exercise intensity) during pedaling. Since the end of the 1980s, these instruments have been used to monitor training, to perform field-based performance tests, to analyze cycling competitions, and to evaluate changes in equipment. They can be classified according to their location on the bike (e.g., rear hub, crank, chainring, pedal, shoe, or handlebar) or to the technology used (e.g., strain gauges, accelerometers, or multi-sensors to measure wind-speed, slope, etc.) [1].

Nowadays, numerous authors have assessed the validity and reliability of the power output measurements of several powermeters (e.g., Stages, Garmin Vector, Quarq, Keo Power, etc.) [2]. However, this is not the case for two widely used devices in the scientific literature [1,3,4,5]: Power2Max (strain gauges in the chainring) and PowerTap G3 (strain gauges in the rear hub). While their validity and reliability during submaximal pedaling in a seated position has been proven [3,5], the validity and reliability of the PowerTap during supramaximal pedaling (i.e., 5 s sprints) and under different pedaling conditions (i.e., seated vs. standing cyclists’ positions, low vs. high gear ratios used) has been questioned [3,4]. To the best of our knowledge, no previous study has analyzed Power2Max validity and reliability under these types of efforts and conditions.

Furthermore, a recent case study that registered data during competition [1] showed that these two portable powermeters were not interchangeable for short and high-intensity efforts (i.e., < 10 s and > 7.5 W·kg^−1^, respectively). This is crucial, because the ability to repeat these efforts is a differential factor between elite and non-elite male and female cyclists, and also because most of these studies obtained data from different types of portable powermeters [6,7,8].

Therefore, the main purpose of the present study was to compare the concurrent validity of two portable powermeters (PowerTap vs. Power2Max) to measure different types of efforts (submaximal, maximal, and supramaximal) in cycling. The reliability of these devices during submaximal and supramaximal efforts was also analyzed.

## 2. Materials and Methods

Ten club cyclists of performance level 3 (1–5 scale [9]) participated in this study (age: 22.1 ± 8.1 years, height: 1.79 ± 0.06 m, body mass: 67.5 ± 3.3 kg; maximal aerobic power: 352.6 ± 44.6 W; cycling experience: 6.6 ± 4.0 years). All of them voluntarily participated and signed written consent. Inclusion criteria were to have competed in cycling for at least 2 years and to have a training volume of more than 3000 km before the start of the study. The study was approved by the University Ethics Committee and met the requirements of the Declaration of Helsinki for research on human beings.

### 2.1. Procedures

The testing was performed at the beginning of the cyclists’ competitive season (February–April). They arrived at the laboratory (800 m of altitude) with their bikes, after a 48 h period without hard training. First, the cyclists’ anthropometrical characteristics and the bikes’ dimensions were registered [10]. The bikes’ dimensions and the clipless pedals were then replicated in a carbon fiber bike (Scott Adicct 30, Scott Sports, Givisiez, Switzerland) where one cadence sensor and two powermeters were installed. The comparison between the registry of these devices allowed the study of the concurrent validity. This bike was set on a cyclosimulator (Cateye CS-1000, Cateye Co., Ltd., Osaka, Japan) [11], and the cyclists performed a 10 min 100 W warm-up period with a 5 min rest before starting the test.

**Figure 1:**
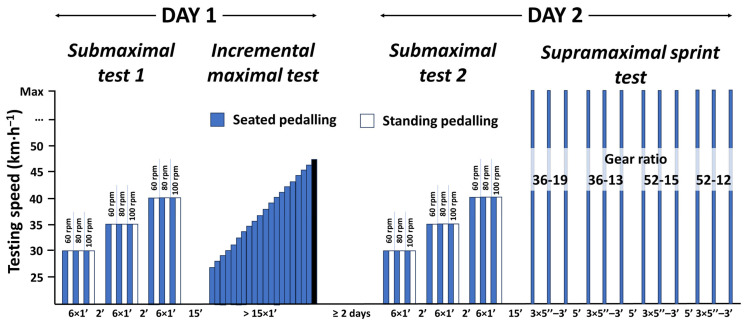
Schematic representation of the experimental design of the present study. Max = maximum possible speed; rpm = revolutions per minute; Gear ratio = Number of chainring teeth-number of sprocket teeth.

The assessment protocol (Figure 1) was performed in two sessions (day 1 and day 2) separated by a minimum of 48 h, and under similar environmental conditions (20–25 °C, 60–65% relative humidity). On the first day, a submaximal test (submaximal test 1) and an incremental maximal test were performed, with a 15 min rest between them. On the second day, another submaximal test (submaximal test 2) and a supramaximal sprint test were performed, with a 15 min rest between them. The sub-maximal tests were used to analyze the inter-day reliability while the supra-maximal sprint tests were used to analyze intra-day reliability. The riding position was standardized, with the cyclists having to rest their hands on the brakes while holding a seated position during both the incremental and supramaximal tests. During the submaximal test, the cyclists pedaled while both seated and standing. In all tests, they were able to drink water at libitum during the recovery periods to avoid dehydration.

#### 2.1.1. Cadence Sensor and Powermeters’ Adjustment

The cadence sensor (Garmin GSC 10, Lenexa, KA, USA) was placed at the right chainstay, and the two powermeters were placed at the chainring (Power2Max Type S, Waldhufen, Germany) and at the rear hub (PowerTap G3, Madison, WI, USA). These three devices were paired with three measurement units (Garmin, Lenexa, KA, USA): Edge 810, Edge 500, and Edge 705, respectively. They were configured at 1 Hz sample frequency and installed on the handlebar stem. To avoid the effect of temperature on the calibration procedure, the bike remained at the same ambient conditions in which the data were obtained for at least 30 min before the start. Afterwards, the power meters were zeroed according to the manufacturer’s guidelines before performing each test [1]. The rear wheel pressure was also standardized at 7 atmospheres.

#### 2.1.2. Submaximal Tests (1 and 2)

On days 1 and 2, three sets of pedaling at 30, 35, and 40 km·h^−1^ were performed, with 2 min rest between them. Each set consisted of 6 repetitions of 1 min pedaling at different cadences (60, 80, and 100 rpm) and riding positions (seated and standing, alternatively). The cyclists adapted their position and cadence during the first 30 s of each repetition, and the data from the last 30 s were used for the analysis. The data from day 1 were used to analyze the concurrent validity, and the data from day 2 were used to analyze the inter-day reliability. The cadence sensor display (i.e., Edge 810) was used by the cyclists as visual reference to adapt their cadence, and the cyclosimulator display (Cateye CS-1000) to adapt their speed.

#### 2.1.3. Incremental Maximal Test

On day 1, this test was performed following the submaximal test, after a recovery period of 15 min. The initial testing speed was set at 27 km·h^−1^, increasing by 1 km·h^−1^ every minute until the cyclist was not able to maintain the speed [11]. The gear ratio was freely chosen by the cyclists, and they were asked to maintain a cadence between 85 and 100 rpm and to remain seated at all times during the test. Data from the entire minute was used for the analysis, and the display of the cyclosimulator (Cateye CS-1000) was used as visual reference for the cyclists to adapt to the testing speed.

#### 2.1.4. Supramaximal Sprint Test

On day 2, the cyclists performed this test after the submaximal test, following a 15 min recovery period. It consisted of 4 sets of seated pedaling at supramaximal intensity with a 4 min rest in between. Each set consisted of 3 repetitions of 5 s supramaximal pedaling using different gear ratios (36-19, 36-13, 52-15 and 52-12) with 3 min of active pedaling at 100 W between each repetition. The test started at a cadence of 80 rpm after a 10 s countdown. At the end of the recovery period, another 10 s countdown was displayed, and the cyclists were instructed to pedal as fast as possible for another 5 s. The peak values of power and cadence measurements during the entire 5 s period were analyzed, and the Power2Max display (i.e., Edge 500) was used as a visual reference by the cyclists to adapt their power and cadence during the recovery period.

### 2.2. Statistical Analysis

Data of the three measurement units (Edge 810, Edge 500, and Edge 705) were analyzed using the same cycling software (Golden Cheetah 3.1 [12]). The results are expressed as mean ± SD. The SPSS+ software was used to analyze both the power and cadence measurements (v.26.0, IBM Corp, Armonk, NY, USA). One-way analysis of variance (ANOVA) for repeated measures was applied to compare the two powermeters (PowerTap vs. Power2Max) when additional variables were not included. Two- and three-way ANOVA for repeated measures were used to analyze the effects of testing speed (30, 35, and 40 km·h-1), cadence (60, 80, and 100 rpm) and pedaling position (standing vs. seated) on the percentual differences between the two powermeters. These percentual differences were calculated as follows: Differences (%) = (PowerTap − Power2Max) × 100/mean value of the two powermeters. When a significant F value was found, the Newman–Keuls post hoc analysis was used to establish statistical differences between means, and the 95% confidence interval (CI95%) of these differences was calculated. Pearson correlation coefficient (r) was used to assess the relationships between variables. Inter- and intra-day reliability of both submaximal and supramaximal sprint tests was assessed using the coefficient of variation (CV) and the intraclass correlation coefficient (ICC) [13,14]. Assumptions of the ANOVA and Pearson correlation tests were examined prior to the analysis. Values of *p* < 0.05 were considered statistically significant.

## 3. Results

### 3.1. Concurrent Validity

#### 3.1.1. Submaximal Test (Day 1)

Significant correlations (*p* < 0.001) were found between the power and cadence data registered by the two powermeters (r = 0.992 and 0.998, respectively). Table 1 shows that there were significant differences between PowerTap and Power2Max in the power (−0.7 ± 2.8%; CI95% = −1.1 and −0.2%; F = 9.4 and *p* = 0.003) and cadence measurements (−1.4 ± 1.3%; CI95% = −1.6 and −1.3%; F = 255.8 and *p* < 0.001). The power differences did not depend on the testing speed, cadence, and pedaling position (F = 1.0, 0.4, and 0.2; *p* = 0.36, 0.65, and 0.70, respectively). On the contrary, the cadence differences depended on the cadence values and the combination of this variable with the pedaling position (F = 19.3 and 6.7; *p* = 0.000 and 0.002, respectively), being higher at lower cadences in the standing position.

**Figure 2:**
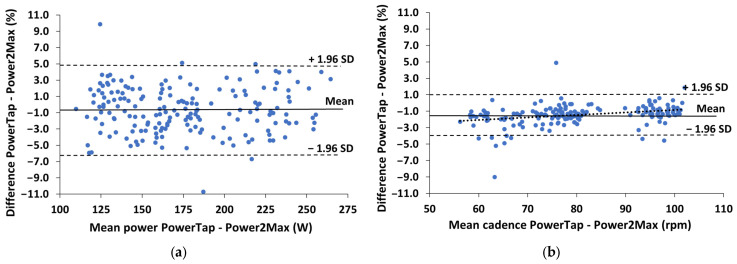
Bland–Altman plots of the differences between the PowerTap and Power2Max measurements during the submaximal maximal test: (**a**) Percentual differences in the power measurements; (**b**) Percentual differences in the cadence measurements. The short-dashed lines represent the upper and lower 95% limits of agreement (±1.96 SD), the solid line represents the bias (Mean) and the dotted line represents the tendency (correlation) between the two variables.

Figure 2 shows the Bland–Altman plots of the percentual differences in both power and cadence measurements between the two powermeters during the submaximal test (Day 1). The power differences were constant (r = −0.01 and *p* > 0.05), while the cadence differences decreased as cadence increased (r = 0.31 and *p* < 0.001).

#### 3.1.2. Incremental Maximal Test

**Figure 3:**
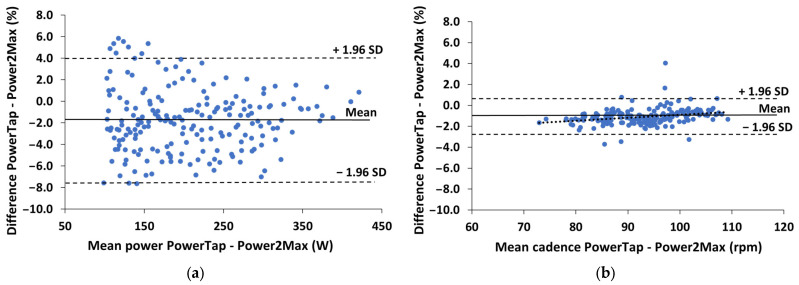
Bland–Altman plots of the differences between the PowerTap and Power2Max measurements during the incremental maximal test: (**a**) Percentual differences in the power measurements; (**b**) Percentual differences in the cadence measurements. The short-dashed lines represent the upper and lower 95% limits of agreement (±1.96 SD), the solid line represents the bias (Mean) and the dotted line represents the tendency (correlation) between the two variables.

Significant correlations (*p* < 0.001) were found between the power and cadence measurements registered by the two powermeters (r = 0.997 and 0.996, respectively). Table 2 shows that there were significant differences between PowerTap and Power2Max in the power (−1.8 ± 2.9%; CI95% = −2.2 and −1.4%; F = 84.5 and *p* < 0.001) and cadence (−1.1 ± 0.8%; CI95% = −1.2 and −1.0%; F = 414.6 and *p* < 0.001). The power differences did not depend on the speed (F = 0.22 and *p* = 1.0), although a tendency of decreasing while intensity increased was observed (Figure 3). On the contrary, the cadence differences depended on testing speed (F = 0.84 and *p* = 0.67), being its highest at the lowest cadences (Figure 3).

Figure 3 shows the Bland–Altman plots of the percentual differences in both power and cadence measurements between the two powermeters during the incremental maximal test. The power differences were constant (r = −0.01 and *p* > 0.05), while the cadence differences decreased as cadence increased (r = 0.32 and *p* < 0.001).

#### 3.1.3. Supramaximal Sprint Test

A significant relation (r = 0.850 and *p* < 0.001) was found between the peak power registered by the two powermeters. However, the correlation was lower and negative for the peak cadence (r = −0.253 and *p* < 0.05). The peak cadence measurements of the Power2Max correlated positively (r = 0.635 and *p* < 0.001) with the cadence sensor (Garmin GSC 10, gold standard) while PowerTap correlated negatively (r = −0.427 and *p* < 0.001). Table 3 shows that there were significant differences between PowerTap and Power2Max in both power (1.7 ± 9.3%; CI95% = 0.2 and 4.4%; F = 4.1 and *p* < 0.05) and cadence measurements (−11.4 ± 14.0%; CI95% = −13.6 and −8%; F = 66.3 and *p* < 0.001). There were also significant differences between these two powermeters and the cadence sensor (F = 80.5 and 7.1, *p* = 0.000 and 0.009, respectively). The power differences between the two powermeters depended on the gear ratio used (F = 2.7 and *p* < 0.05), being 12.5 ± 41.9%, −3.9± 8.9%, 3.5 ± 4.4%, and 6.1 ± 3.1% for the gear ratios of 36-19, 36-13, 52-15, and 52-12, respectively. The cadence differences between the two powermeters also depended on the gear ratio used (F = 22.9 and *p* < 0.001), being lower at higher gear ratios (−36.8 ± 40.9%, −43.1 ± 17.8%, −8.1 ± 14.6%, and 1.3 ± 1.8% for the gear ratios of 36-19, 36-13, 52-15, and 52-12, respectively).

**Figure 4:**
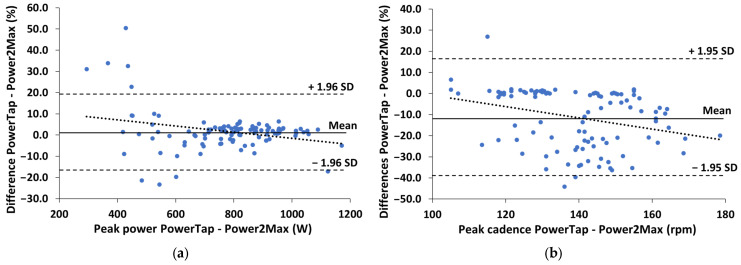
Bland–Altman plots of the differences between the PowerTap and Power2Max measurements during the supramaximal sprint test: (**a**) Percentual differences in the peak power measurements; (**b**) Percentual differences in the peak cadence measurements. The short-dashed lines represent the upper and lower 95% limits of agreement (±1.96 SD), the solid line represents the bias (Mean) and the dotted line represents the tendency (correlation) between the two variables.

Figure 4 shows the Bland–Altman plots of the percentual differences in both peak power and peak cadence measurements between the two powermeters during the supramaximal sprint test. Power and cadence differences increased as cadence increased (r = −0.34 and −0.37, respectively; *p* < 0.001).

### 3.2. Test–Retest Reliability

Inter-day reliability analysis of the submaximal test showed significant test–retest correlations (*p* < 0.001) for the PowerTap and Power2Max powermeters when analyzing both power (ICC = 0.926 and 0.936, respectively) and cadence measurements (ICC = 0.969 and 0.970, respectively). He test–retest coefficient of variation was different when analyzing the power (5.2 ± 3.9% and 4.6 ± 3.9%, respectively; F = 6.6 and *p* < 0.05) but similar for the cadence measurements (2.4 ± 2.2% and 2.3 ± 2.0%, respectively; F = 0.3 and *p* > 0.05).

Intra-day reliability analysis for the supramaximal sprint test showed overall significant test–retest correlations for the PowerTap and Power2Max powermeters when analyzing both power (ICC between 0.918 and 0.943, and 0.896 and 0.939, respectively; *p* < 0.001) and cadence data (ICC between 0.496 and 0.736, and 0.574 and 0.664, respectively; *p* < 0.05). The reliability of this last variable was highest for the cadence sensor (ICC between 0.987 and 0.994). The power’s coefficient of variation was significantly different in the PowerTap and Power2Max (CV = 4.9 ± 4.3 and 10.0 ± 15.0%, respectively; F = 4.8 and *p* < 0.05). The cadence’s coefficient of variation was higher in the two powermeters compared to the cadence sensor (CV = 6.8 ± 5.6, 6.5 ± 10.6, and 1.1 ± 0.8%, respectively; F = 6.2 and *p* < 0.01).

## 4. Discussion

The main finding of this study is to demonstrate the concurrent validity and reliability of the PowerTap and Power2Max powermeters during the monitoring of both submaximal and incremental maximal efforts (i.e., continuous pedaling), but not during the supramaximal efforts (i.e., sprint pedaling). The small differences observed between the two powermeters during continuous pedaling are justified by their location (i.e., rear hub vs. chainring, respectively) and by the effect of both the cadence and the cyclists’ position on the bike (i.e., higher at lower cadences and in the standing position). On the other hand, the high differences and low reliability of the power and cadence measurements during the sprints are related to the lack of accuracy in the cadence measurements (PowerTap and Power2Max) and to the effect of the gear ratio on the power measurements (PowerTap).

### 4.1. Continuous Pedaling (Submaximal and Incremental Maximal Tests)

The agreement found between PowerTap and Power2Max power measurements during both submaximal and incremental maximal tests (r = 0.992 y 0.997, respectively) is similar to that obtained in previous studies (r = 0.997, *p* < 0.001) in which PowerTap and SRM devices were compared [4]. PowerTap’s underestimation of power relative to Power2Max (Table 1 and Table 2) is justified by their different placement (i.e., rear hub and chainring, respectively). In fact, the power registered in the rear hub is lower than in the chainring, as previously stated by other authors [3,15]. Furthermore, these power differences (between 1.2–3.6 W or 0.7–1.8%) are similar to those obtained in recent studies which compared PowerTap with SRM (1.3 ± 6.0 W at a pedaling intensity between 150 and 350 W) [4] and Power2Max (1–2% at a pedaling intensity between 200 and 315 W) [1]. However, these differences are slightly lower than the 2–4% observed in previous older studies [15,16], probably due to improvements in the bicycle’s equipment over the last few decades (e.g., stiffer carbon fiber cranks and frames), which could decrease the energy lost from the chainring to the rear hub.

The aforementioned differences were not affected by pedaling intensity, cadence, or the cyclist’s position on the bike (Table 1). This is in line with the results of previous studies that analyzed the influence of cadence and the cyclist’s position on power measured by SRM and PowerTap [3], as well as with another study that compared the influence of cadence on power measured using SRM, PowerTap, and Stages [4]. However, the present study shows that cadence measurements were affected by cadence data and the cyclist’s position on the bike (Figure 2b and Figure 3b), being higher at lower cadences in the standing position, which was not investigated in these studies. For this reason, the differences in power during the incremental maximal test tended to decrease when pedaling intensity increased (Figure 3a), since higher pedaling intensity has been associated with higher pedaling cadence [17]. This was not observed during the submaximal test (Figure 2a), probably due to the effect of the cyclist’s position on the bike (i.e., seated vs. standing) and because the cyclists freely chose the gear ratio during this test (please see below the discussion about the influence of the gear ratio).

On the other hand, inter-day reliability of PowerTap and Power2Max was very high for both power (ICC = 0.926 and 0.936, respectively) and cadence measurements (ICC = 0.969 and 0.970, respectively). However, the coefficient of variations of power (CV = 5.2 ± 3.9 and 4.6 ± 3.9%, respectively) were higher than those obtained in previous studies (between 0.8 and 2.8%) [3,4,5]. This could be related to the higher variability needed to adjust the testing speed in the ergometer (i.e., Cateye CS-1000 is an air-braked ergometer in which the cyclists must adjust themselves to the testing speed). Nevertheless, it should be taken into account that the testing variability should not exceed 5%, and that the detectable change in performance represents a magnitude <2% in elite athletes [18], so alternative ergometers must be used in future studies.

### 4.2. Sprint Pedaling (Supramaximal Sprint Test)

The agreement in power measurements between the two devices during the supramaximal sprint test (r = 0.850 and *p* < 0.001) was lower than during continuous pedaling. In addition, PowerTap underestimated power values (mean difference of 1.7 ± 9.3%, CI95% between 0.2 and 4.4%) compared to Power2Max (Table 3). This is in line with previous studies that observed an underestimation of the peak power recorded by PowerTap during supramaximal efforts from 1 to 10 s [1,4], although his was not specifically mentioned by one of them [4]. Furthermore, these differences depended on the gear ratio used, decreasing as pedaling intensity increased (Figure 4a). The higher differences were found at the lowest gear ratio (12.5 ± 41.9% and 36-19, respectively) qualitatively similar to what was observed in a previous study that only investigated the effect of gear ratios 39-23, 39-17, and 39-14 [3]. These authors found that PowerTap underestimated the power data compared to SRM, but the methodology of the sprint tests was different, and the pedaling cadence was lower than in the present study (please see below). Additionally, in the present study, the power differences were smaller at higher gear ratios, which is consistent with the results from another study that investigated the effect of gear ratios of 53-19, 53-17, and 53-15 [4]. Therefore, it seems clear that PowerTap does not correctly measure power during supramaximal efforts when using different gear ratios, as previous studies have reported [3].

Additionally, Power2Max showed low intra-day reliability to measure peak power, with a coefficient of variation higher than 5% and that observed in PowerTap (CV = 10.0 ± 15.0 and 4.9 ± 4.3%, respectively). Both devices use four strain gauges and internally calculate the pedaling cadence, so the sources of error in power measurements can be diverse [4]. Cadence measurement could be one of them, because cadence is used to calculate power (i.e., Power = torque × crank angular velocity). Thus, our results surprisingly showed that the agreement between the two devices for measuring cadence is very weak (r = −0.253 and *p* < 0.05). Moreover, PowerTap and Power2Max underestimated peak cadence measurements compared to the cadence sensor (Table 3), showing a weak agreement with this device (r = −0.427 and 0.635, respectively) and higher coefficients of variation (CV = 6.8 ± 5.6, 6.5 ± 10.6, and 1.1 ± 0.8%, respectively). Along the same lines, a recent study that compared SRM and Favero showed that the CVs of these two devices during sprint pedaling were higher than those observed during continuous pedaling for both power and cadence measurements [18]. An important difference of the present study with respect to previous studies [3,4,18] is that the cyclists were already pedaling before the start of the sprint tests, thus resulting in higher cadence values (i.e., >160 rpm and 130–150 rpm, respectively). Taking into account that the accuracy of cadence measurement with portable powermeters depend on the cadence value (Figure 4b), this should be considered during pedaling efforts with cadences higher than 150 rpm (e.g., track cycling or sprints in road cycling).

### 4.3. Limitations

The main limitations of the present work were the following: (A) The tests were performed under laboratory conditions, which does not represent the changes in the environmental temperature and vibrations that affect the power output measured [4]. However, the laboratory tests allowed us to better standardize the protocols and identify the variables that affected the powermeter measurement (i.e., type of effort, position on the bike or cadence). (B) Only one measurement unit of PowerTap and Power2Max were used, knowing that it is possible to obtain differences between units of the same powermeter [5,19]. (C) Inter-day reliability was analyzed in the submaximal test, while intra-day reliability was analyzed in the supramaximal sprint test. This was due to the need to design a cost-effective protocol for competitive cyclists, including no more than two days of testing. Future studies should design a protocol to analyze inter- and intra-day reliability in both the submaximal and supramaximal sprint tests. (D) The reliability (CVs) of the power and cadence measurements during each condition depended both on the reliability of the devices (PowerTap and Power2Max) and on the biological variability of the cyclists while pedaling (i.e., having to adjust the velocity on the ergometer), so alternative ergometers to the Cateye CS-1000 should be used in future studies. (E) The minimum sample size for adequate statistical power to analyze test–retest reliability was not calculated [20], although the sample size of the present study was similar to those used in previous recent studies on the same topic [21,22,23]. (F) The participants of the study had a performance level 3 (i.e., club competitors). Therefore, future studies should check if these results are similar in other levels of performance (e.g., 1 and 5).

## 5. Conclusions

The PowerTap and Power2Max powermeters are valid and reliable devices for measuring power and cadence during continuous cycling efforts at pedaling intensities between ~100 and 450 W. In these type of efforts, PowerTap underestimates power by 0.7–1.8%, depending on pedaling cadence, the cyclist’s position on the bike (seated vs. standing), and the gear ratio used.

However, the validity and reliability of these powermeters for monitoring power and cadence during sprint efforts (>500 W) is highly questionable, as they are affected by the gear ratio used (PowerTap) and by their lack of accuracy in cadence recording (PowerTap and Power2Max), among other possible factors that should be investigated in further studies.

From a practical perspective, power and cadence recordings obtained during high-intensity pedaling efforts with portable powermeters should be interpreted with caution in both training and scientific contexts. Coaches and cyclists should be careful with data results from training and competition sprints (>500 W). Additionally, the cadence measurement of these devices should be improved because of its possible effect on power measurement accuracy.

## Figures and Tables

**Table 1:** Mean ± SD of the power and cadence registered by the two powermeters (PowerTap vs. Power2Max) during the submaximal test (Day 1) at different speeds (30, 35, and 40 km·h^−1^), cadences (60, 80, and 100 rpm) and pedaling positions (standing and seated).

Speed	Cadence	Pedaling	Power (W)		Cadence (rpm)	
(km·h^−1^)	(rpm)	Position	PowerTap	Power2Max	PowerTap	Power2Max
30	60	Standing	134.9 ± 9.5	135.6 ± 7.9	59.5 ± 2.4	60.7 ± 2.6
		Seated	141.0 ± 6.7	141.7 ± 7.2	60.5 ± 1.9	61.4 ± 2.0
	80	Standing	138.2 ± 9.7	139.5 ± 11.9	75.2 ± 2.5	75.5 ± 2.6
		Seated	130.8 ± 8.8	130.3 ± 11.4	80.5 ± 2.5	80.3 ± 4.0
	100	Standing	125.4 ± 9.9	125.0 ± 7.8	95.8 ± 2.6	97.1 ± 2.6
		Seated	126.5 ± 9.0	126.9 ± 9.9	97.6 ± 1.7	99.0 ± 1.5
35	60	Standing	163.0 ± 10.7	164.5 ± 10.1	65.2 ± 3.7	67.2 ± 3.5
		Seated	170.5 ± 9.2	173.2 ± 9.2	64.6 ± 2.3	65.8 ± 2.4
	80	Standing	169.5 ± 12.3	169.8 ± 10.2	76.3 ± 1.5	76.9 ± 1.4
		Seated	172.3 ± 9.1	174.6 ± 10.1	78.5 ± 1.6	79.8 ± 1.5
	100	Standing	164.2 ± 11.2	165.9 ± 11.3	95.7 ± 3.7	96.3 ± 2.8
		Seated	170.0 ± 13.2	171.5 ± 13.1	95.8 ± 3.2	97.0 ± 2.9
40	60	Standing	207.4 ± 12.8	209.5 ± 12.0	72.1 ± 3.1	73.9 ± 3.0
		Seated	217.4 ± 20.9	218.5 ± 15.1	70.9 ± 4.4	72.5 ± 3.3
	80	Standing	219.7 ± 13.0	219.4 ± 13.8	77.8 ± 1.3	78.5 ± 2.1
		Seated	227.5 ± 13.6	228.9 ± 7.4	79.5 ± 2.6	80.6 ± 2.3
	100	Standing	225.4 ± 21.0	227.0 ± 19.3	94.8 ± 3.4	95.4 ± 3.3
		Seated	243.6 ± 15.1	246.2 ± 13.3	97.7 ± 2.1	98.7 ± 2.0
		Overall	174.8 ± 40.0	176.0 ± 40.1 *	79.9 ± 13.3	81.0 ± 13.2 *

Overall: Mean of all speeds, cadences, and positions for the same powermeter. * Significant differences (*p* < 0.05) between the two powermeters.

**Table 2:** Mean ± SD of the power and cadence registered by the two powermeters (PowerTap vs. Power2Max) during the incremental maximal test at different speeds (from 27 to 49 km·h^−1^).

Speed	Cyclists	Power (W)		Cadence (rpm)	
(km·h^−1^)	(n)	PowerTap	Power2Max	PowerTap	Power2Max
27	10	105.3 ± 5.1	106.4 ± 4.3	85.9 ± 6.8	86.8 ± 6.7
28	10	111.3 ± 5.5	112.0 ± 6.3	88.1 ± 9.0	88.7 ± 8.7
29	10	119.5 ± 4.8	121.4 ± 4.6	88.8 ± 8.1	89.7 ± 8.0
30	10	127.8 ± 4.8	130.1 ± 4.4	88.7 ± 6.9	89.8 ± 6.6
31	10	136.5 ± 4.9	139.0 ± 4.3	89.0 ± 4.9	90.3 ± 5.0
32	10	143.9 ± 6.8	147.2 ± 4.4	90.4 ± 4.7	91.4 ± 4.5
33	10	154.9 ± 6.9	157.0 ± 6.4	90.7 ± 7.0	91.4 ± 6.7
34	10	164.2 ± 7.7	167.4 ± 5.4	88.8 ± 6.7	90.1 ± 6.9
35	10	177.3 ± 5.3	180.4 ± 5.3	90.8 ± 5.4	92.2 ± 5.4
36	10	187.8 ± 9.3	191.6 ± 7.0	92.0 ± 6.8	93.1 ± 6.5
37	10	199.8 ± 7.6	203.1 ± 7.2	93.6 ± 7.2	94.5 ± 7.2
38	10	213.8 ± 8.7	216.7 ± 9.0	95.1 ± 8.6	96.0 ± 8.3
39	10	224.7 ± 13.0	229.0 ± 10.0	95.4 ± 7.7	96.7 ± 7.6
40	10	236.9 ± 11.2	242.4 ± 9.4	94.7 ± 4.5	95.9 ± 4.6
41	10	251.1 ± 12.7	255.6 ± 10.0	95.9 ± 5.0	96.6 ± 4.7
42	10	270.2 ± 16.9	275.4 ± 13.8	96.0 ± 5.1	97.1 ± 4.9
43	10	286.5 ± 24.3	291.9 ± 21.6	96.6 ± 5.8	97.8 ± 5.7
44	10	301.7 ± 26.2	307.2 ± 24.9	97.5 ± 7.9	98.5 ± 7.6
45	9	311.7 ± 17.1	320.0 ± 9.7	99.1 ± 8.3	99.9 ± 8.3
46	6	339.9 ± 33.5	344.5 ± 26.7	99.5 ± 7.4	100.0 ± 7.7
47	4	380.1 ± 43.4	382.2 ± 38.3	98.1 ± 7.2	99.0 ± 7.0
48	1	371.9	376.8	91.4	92.8
49	1	385.0	390.8	92.8	94.2
	Overall	205.2 ± 75.2	208.8 ± 76.2 *	92.8 ± 7.5	93.8 ± 7.4 *

Overall: Mean of all speeds for the same powermeter. * Significant differences (*p* < 0.05) between the two powermeters.

**Table 3:** Mean ± SD of the peak power and peak cadence registered by the two powermeters (PowerTap vs. Power2Max) and the cadence sensor (Garmin GSC10) during the supramaximal sprint test performed with different gear ratios (36-19, 36-13, 52-15, and 52-12).

Gear	Sprint	Power (W)		Cadence (rpm)		
Ratio	Number	PowerTap	Power2Max	PowerTap	Power2Max	Garmin GSC10
36-19	1	502.8 ± 86.7	415.9 ± 149.6	108.8 ± 23.6	163.4 ± 37.6	184.1 ± 27.2
	2	561.4 ± 103.0	630.2 ± 104.0	107.6 ± 10.5	173.2 ± 40.2	195.5 ± 17.4
	3	541.6 ± 79.6	442.8 ± 185.4	106.0 ± 16.1	153.1 ± 57.8	195.1 ± 15.4
36-13	1	759.8 ± 85.8	831.4 ± 199.5	118.2 ± 18.6	177.6 ± 14.6	177.7 ± 4.9
	2	769.6 ± 71.1	783.1 ± 88.1	116.4 ± 21.7	180.0 ± 10.3	179.0 ± 4.9
	3	800.6 ± 54.2	824.3 ± 65.4	115.7 ± 24.9	180.0 ± 5.2	179.6 ± 4.2
52-15	1	882.7 ± 105.7	862.9 ± 108.1	139.4 ± 12.0	151.6 ± 6.5	152.9 ± 6.7
	2	917.1 ± 112.9	887.7 ± 146.1	143.1 ± 20.7	152.1 ± 5.4	154.3 ± 6.6
	3	910.9 ± 75.0	872.3 ± 84.1	141.0 ± 17.7	152.7 ± 7.1	153.7 ± 5.5
52-12	1	928.1 ± 115.8	872.9 ± 119.8	127.0 ± 5.6	124.1 ± 4.8	125.7 ± 5.8
	2	896.2 ± 107.8	836.3 ± 122.0	125.0 ± 5.5	122.8 ± 5.5	123.9 ± 6.0
	3	937.7 ± 99.7	894.8 ± 104.9	126.6 ± 6.0	126.9 ± 5.0	127.3 ± 6.6
Overall		784.0 ± 179.2	762.9 ± 204.9 *	122.9 ± 20.2 #	154.8 ± 30.6 #	162.4 ± 27.7

Overall: Mean of all gear ratios for the same powermeter or cadence sensor. * Significant differences (*p* < 0.05) in the power measurements between the PowerTap and Power2Max. # Significant differences (*p* < 0.001) in the cadence measurements with respect to the cadence sensor (Garmin GSC10).

## Data Availability

Data are contained within the article.
